# The Contribution of Forensic Medical Investigations in Road Accident Deaths

**DOI:** 10.7759/cureus.81166

**Published:** 2025-03-25

**Authors:** Matteo Antonio Sacco, Saverio Gualtieri, Chara Spiliopoulou, Alessandro Pasquale Tarallo, Isabella Aquila

**Affiliations:** 1 Department of Medical and Surgical Sciences, Magna Graecia University, Catanzaro, ITA; 2 Department of Forensic Medicine and Toxicology, School of Medicine, National and Kapodistrian University of Athens, Athens, GRC; 3 Department of Medical and Surgical Sciences, Institute of Legal Medicine, Magna Graecia University, Catanzaro, ITA

**Keywords:** alcohol, autopsy, forensic sciences, road traffic accidents, substance abuse

## Abstract

Forensic medicine plays a crucial role in investigating fatalities resulting from road accidents, particularly in assessing physical injuries and toxicological findings to determine their correlation with accident dynamics. The expertise of forensic pathologists is essential not only in establishing the cause of death but also in reconstructing the events leading to the fatal outcome and identifying the potential influence of alcohol or psychoactive substances. The presence of such substances in road accident victims is a well-documented factor contributing to impaired driving, making forensic toxicology a key component in post-mortem examinations.

When road accidents result in fatalities, forensic investigations, especially autopsies, provide critical insights into the cause of death and assist in determining any legal responsibilities. In many cases, judicial authorities mandate forensic examinations to clarify whether death resulted directly from the accident or was influenced by other factors such as pre-existing medical conditions or substance intoxication. This study reviews existing research on forensic investigations in road accidents, emphasizing their role in enhancing road safety measures and ensuring legal accuracy.

## Introduction and background

Global road traffic accidents

Road accidents are among the most significant public health challenges globally, with a profound social and economic impact. According to the World Health Organization (WHO), road accidents are the ninth leading cause of death worldwide among adults and the leading cause of death among young people aged 15 to 19 years [1]. Among children aged 10 to 14 years and young adults aged 20 to 24 years, they represent the second leading cause of death. The global burden of road accidents is expected to rise further, becoming the third leading cause of death and disability by 2030 if adequate measures are not taken [2]. This trend is particularly alarming in low- and middle-income countries, which account for the majority of road fatalities due to a combination of inadequate road infrastructure, weak law enforcement, and limited access to emergency medical services.

The burden of road accidents is not evenly distributed among countries, revealing stark inequalities between high-income and low-income regions. Vulnerable road users such as pedestrians, cyclists, and motorcyclists are particularly at risk, especially in countries where road safety measures are underdeveloped. Conversely, high-income countries have implemented extensive road safety measures, resulting in a significant reduction in road accident mortality over the past few decades [3]. Despite these improvements, however, road accidents remain a leading cause of death and disability worldwide, emphasizing the need for continued efforts to enhance prevention strategies and promote safer driving behavior.

Road traffic accidents remain a critical global public health issue. According to the WHO, approximately 1.19 million people worldwide lose their lives annually due to road traffic crashes, with an additional 20 to 50 million sustaining non-fatal injuries [4]. Notably, road traffic injuries have become the leading cause of death among children and young adults aged five to 29 years. The burden of these incidents is disproportionately borne by low- and middle-income countries (LMICs), which account for over 90% of global road traffic deaths despite having less than 60% of the world's vehicles [5]. Factors contributing to this disparity include inadequate infrastructure, limited enforcement of traffic laws, and insufficient access to post-crash emergency care. In high-income countries, concerted efforts have led to a decline in road traffic fatalities. For instance, the United States experienced a 25% reduction in road traffic deaths from 2005 to 2014, attributed to successful interventions such as seat belt laws, enforcement of speed limits, public awareness campaigns on the dangers of impaired driving, and improvements in road and vehicle safety [6].

Recent data indicate that impaired driving remains a significant concern. In 2022, approximately 30% of young drivers aged 15 to 20 years who were fatally injured in crashes had blood alcohol concentrations (BACs) of 0.01 g/dL or higher [7]. Additionally, among US high school students who drove, 5.4% reported driving after consuming alcohol at least once in the past 30 days [4]. These statistics underscore the ongoing challenges in preventing underage drinking and driving. This behavior significantly increases the risk of accidents and highlights the persistent challenge of reducing alcohol-related road fatalities [5].

Road safety: European context

In the European context, road accidents continue to represent a substantial public health problem, with significant variations in mortality and injury rates between different countries. The WHO European Status Report on Road Safety, in 2018, estimated that approximately 120,000 people die annually in road accidents across the European region, while 2.4 million suffer non-fatal injuries [1]. Vulnerable road users, such as pedestrians, cyclists, and motorcyclists, make up nearly 40% of these fatalities [1].

The economic burden of road accidents in Europe is also substantial, with costs averaging more than 3% of each country’s gross domestic product (GDP) [1]. Eastern European nations report significantly higher road mortality rates compared to Nordic countries, largely due to differences in infrastructure quality, enforcement of traffic regulations, and road safety measures. Pedestrians in these regions are particularly vulnerable, as urban planning and pedestrian protection policies may not be as developed as in Western and Northern Europe. Similarly, Southern European countries such as Italy, Greece, Malta, and France exhibit some of the highest fatality rates for motorcycle accidents. This trend reflects the widespread use of motorcycles in these regions, coupled with variations in helmet usage, road conditions, and enforcement of safety measures [1].

Preventive measures such as the use of seat belts and helmets have proven highly effective in reducing road accident fatalities. Research indicates that wearing a seat belt lowers the risk of death by 40% to 65% for front-seat occupants and by 25% to 75% for rear-seat passengers [6]. Similarly, wearing a helmet reduces the risk of fatal head injuries by 40% and the risk of serious head injuries by 70%. Despite these well-documented benefits, compliance with safety regulations remains inconsistent across Europe, particularly in countries with less rigorous enforcement policies, like Bulgaria, Romania, and Poland, where seat belt usage rates are lower compared to Western European nations [6].

## Review

Materials and methods

A comprehensive literature search was conducted using PubMed and Scopus, covering studies published between January 2010 and December 2024. The search terms included “alcohol OR psychotropic drugs OR autopsy” AND “road-traffic accidents”, applied to titles, abstracts, and keywords to maximize relevant results. Only peer-reviewed journal articles were considered, while conference papers, reports, and gray literature were excluded to maintain scientific rigor. The search was restricted to English-language publications to ensure accessibility and consistency in data interpretation.

During the selection process, studies were screened based on their relevance to forensic investigations, toxicological findings, and public health implications. A total of 15 studies met the inclusion criteria, categorized as follows: five forensic investigations involving autopsies of accident victims, five clinical studies analyzing the effects of alcohol and drugs on driving behavior, and five public health research studies focusing on prevention strategies and rehabilitation programs.

Out of approximately 400 initially identified studies, many were excluded due to insufficient data, lack of relevance, or methodological limitations. To enhance the comprehensiveness of the review, reference lists of the selected studies were cross-checked to identify additional relevant research. This approach ensured that the final selection encompassed a broad and methodologically sound representation of the available literature on the role of alcohol, narcotics, and psychoactive substances in road traffic accidents. To assess the methodological quality of the included studies, a risk of bias assessment was conducted using the Cochrane Risk of Bias tool for randomized studies and the Newcastle-Ottawa Scale for observational studies. Each study was independently assessed by two reviewers, and any discrepancies were resolved through discussion. The evaluation considered factors such as selection bias, information bias, confounding variables, and outcome assessment. Studies with high risk of bias due to small sample sizes, lack of control groups, or incomplete data reporting were identified, and their limitations were noted in the discussion. No statistical analyses were conducted as this study is a qualitative review of existing literature. The findings presented are based on the synthesis of previously published research rather than primary data analysis.

Results and discussion

The Role of Autopsy

A comprehensive review of forensic medical investigations in road accident deaths necessitates an understanding of key forensic methodologies. Autopsies serve as a primary tool in differentiating between natural deaths, accidental deaths, and homicides in traffic-related cases. By examining injury patterns, forensic experts can determine factors such as seatbelt use, speed of impact, and pre-existing medical conditions that may have contributed to fatal outcomes. In different countries, forensic investigative methodologies vary significantly [8,9]. In some nations, such as Sweden and Finland, forensic autopsies are legally mandated for all road accident fatalities to determine the precise cause of death and assess potential contributing factors, including alcohol or drug impairment [10,11]. In contrast, countries like the United Kingdom and the United States only require forensic autopsies under specific circumstances, such as suspected foul play, unclear cause of death, or cases involving minors. Additionally, the threshold for toxicological screening varies, with some jurisdictions performing routine post-mortem toxicology tests, while others conduct them selectively based on initial forensic assessments. These differences in methodology can impact the consistency of forensic data used for legal, medical, and public health interventions. The availability of advanced forensic tools and imaging techniques also differs, impacting the depth of analysis and the accuracy of conclusions. Standardizing forensic practices internationally could enhance the comparability of data and improve road safety measures globally.

Post-mortem Toxicological Investigations

Toxicological analysis is another essential aspect, identifying the presence of alcohol, drugs, or other substances that may have impaired the driver's or pedestrian's ability to react appropriately. Studies indicate that a significant proportion of road accident fatalities involve substance influence, emphasizing the importance of forensic testing in corroborating or refuting legal claims [12]. For instance, a comprehensive analysis conducted in Galicia, Spain, over a decade (2009-2019) revealed that psychotropic substances were detected in nearly 40% of the 710 traffic accident victims examined. Ethyl alcohol was the most frequently identified substance, followed by benzodiazepines and cocaine [12].

The analysis of six selected studies revealed that alcohol, cannabis, and benzodiazepines are the most commonly detected substances in road accident fatalities. Among these, alcohol is the leading contributor, found in nearly 25% of cases involving fatal road accidents [9]. Its role in impairing cognitive and motor functions is well-documented, significantly increasing the likelihood of crashes and exacerbating the severity of injuries sustained. The correlation between BAC levels and fatal outcomes is well-documented, particularly in high-speed collisions and single-vehicle crashes. However, the legal BAC limit varies across countries, ranging from 0.02% in some Scandinavian nations to 0.08% in countries like the United States and the United Kingdom. Despite these differences, exceeding the respective legal limit significantly increases the risk of severe and fatal accidents [10]. Drivers under the influence of alcohol exhibit slower reaction times, reduced peripheral vision, impaired decision-making abilities, and a tendency to engage in risky behavior, such as excessive speeding and failing to wear seat belts [11].

Studies have shown that cannabis is frequently detected in traffic accident cases involving drug testing [13]. For instance, a study reported that on weekends, approximately 26-27% of individuals involved in car crashes tested positive for cannabis [13]. Its psychoactive component, tetrahydrocannabinol (THC), is known to impair coordination, concentration, and reaction time, all of which are essential for safe driving [14]. Studies have demonstrated that the presence of THC in the bloodstream correlates with an increased risk of motor vehicle collisions, especially when combined with alcohol or other substances [13]. In many cases, cannabis use is associated with prolonged reaction times and decreased awareness of external stimuli, leading to poor vehicle control and delayed responses to hazardous situations.

Benzodiazepines, widely prescribed for anxiety, insomnia, and other medical conditions, have been detected in a significant proportion of road accident victims in three studies. For example, a study analyzing 3,147 road accident fatalities found benzodiazepines present in the blood of 8% of the cases [15]. Another study reported that 14% of injured drivers tested positive for benzodiazepines [16]. The most commonly detected benzodiazepines in traffic accident cases include diazepam, alprazolam, and clonazepam, all of which have well-documented impairing effects on driving ability [17].

In a study conducted in the Amman district, Jordan, toxicological analysis revealed the presence of alcohol and psychotropic drugs in 36.5% of cases involving fatal road accidents. Benzodiazepines and barbiturates were the most commonly detected substances, while no samples tested positive for cocaine, amphetamines, or illicit cannabis [18]. This finding suggests that, in specific regions, prescription medications may pose a greater threat to road safety than illicit substances. The majority of victims were pedestrians, highlighting the vulnerability of non-motorized road users in high-risk urban environments. Young adults aged 19 to 29 years were disproportionately represented in the sample, confirming the higher risk of fatal accidents in this age group [18].

Another study from the Czech Republic, which analyzed data from the National Register of Autopsies and Toxicological Examinations between 2016 and 2021, found that alcohol was present in 24% of tested cases, with BACs exceeding 0.2 g/kg in many instances [19]. Toxicological tests for substances other than alcohol were conducted in only 49% of cases. Among these, 9% tested positive for addictive substances, including cannabis, benzodiazepines, opioids, and amphetamines. Notably, in several instances, the concentration of these substances reached levels known to cause significant impairment of driving ability, contributing to fatal outcomes. The study underscored the importance of routine toxicological screening in road accident victims to identify potential contributing factors and inform prevention strategies.

In a large-scale study conducted in Spain, toxicological analyses were performed on 710 individuals who died in road accidents over a 10-year period (2009-2019) [17]. Of the 710 cases, 123 involved pedestrians, while 587 were vehicle or motorcycle occupants. Alcohol was the most frequently detected substance, present in 231 cases, predominantly among male victims. Among female victims, benzodiazepines were more commonly detected, followed by alcohol. Benzodiazepines were found in 43 cases, followed by cocaine in 25 cases. Poly-drug use was relatively rare, occurring in 44 cases, with the most common combination being alcohol and cocaine, followed by alcohol and benzodiazepines. Only five cases involved three or more substances simultaneously. These findings highlight the continued prevalence of alcohol as a primary factor in road accident fatalities, despite extensive public awareness campaigns and law enforcement efforts aimed at reducing drink-driving.

Legal and Policy Implications

A comparative analysis of legal frameworks across different countries reveals varying levels of strictness in regulations regarding impaired driving. Some nations enforce zero-tolerance policies for alcohol and drug consumption among drivers, while others implement graded legal limits. The role of forensic investigations in supporting legal proceedings is crucial, particularly in attributing liability in fatal accidents. Strengthening the collaboration between forensic experts and legal professionals can enhance the prosecution of cases involving impaired driving [19].

The effectiveness of punitive measures, such as license suspension and mandatory rehabilitation programs, depends on factors such as the duration of penalties, enforcement consistency, and the presence of repeat offender policies. For example, countries with longer license suspension periods and compulsory rehabilitation programs, such as Sweden and Canada, have reported lower recidivism rates among impaired drivers compared to nations with more lenient approaches [20]. However, some legal systems face challenges in conducting timely toxicological screenings, leading to delays in the judicial process and difficulty in prosecuting offenders effectively.

Sociodemographic and Psychological Considerations

Age, gender, and socioeconomic background influence the prevalence of impaired driving, with notable differences across low-, middle-, and high-income countries. Studies indicate that in high-income countries, impaired driving is more prevalent among young male drivers, particularly in urban areas. In contrast, in low- and middle-income countries, lower enforcement of traffic laws and reduced access to public transportation contribute to higher rates of impaired driving across broader demographic groups [21]. Studies show that young adults and males are overrepresented in fatal accidents involving alcohol and drugs. Psychological factors such as substance dependence, risk-taking behavior, and mental health disorders also play a significant role in impaired driving. Addressing these underlying issues through targeted interventions can improve road safety outcomes [21].

Research indicates that individuals diagnosed with mental health disorders are at a higher risk of being involved in road accidents, particularly when taking prescription medications that impair cognitive function [22]. The interaction between psychotropic drugs and driving ability is a growing concern, necessitating better coordination between mental health professionals and traffic safety authorities.

This raises ethical and legal questions regarding privileged communication between healthcare providers and their patients. In many jurisdictions, physician-patient confidentiality is protected; however, exceptions exist when public safety is at risk. Some countries, such as the United States and Canada, have mandatory reporting laws that require physicians to report individuals whose medical conditions, including severe psychiatric disorders, may compromise their ability to drive safely [22]. In contrast, other nations place the responsibility on the patient to self-report their condition. These differences highlight the ongoing debate on balancing individual privacy rights with public interest in preventing road accidents caused by medically impaired drivers.

Educating drivers and implementing laws about the effects of prescribed medications on their ability to operate a vehicle safely could reduce accident rates.

Implications for Prevention and Road Safety

Findings from forensic investigations contribute to policy recommendations, including enhanced roadside testing and random checks for alcohol and drug impairment, stricter regulations on prescription medications that affect driving abilities, and public awareness campaigns emphasizing the dangers of impaired driving [23]. Technological innovations, such as ignition interlock devices, that prevent intoxicated individuals from operating vehicles are also gaining traction in traffic safety policies [24].

The growing use of artificial intelligence in traffic monitoring presents new opportunities for reducing accidents related to substance impairment [25]. Machine learning algorithms can analyze driver behavior in real time, detecting signs of fatigue, distraction, or impairment [25]. When integrated with vehicle safety systems, these technologies could prevent high-risk drivers from continuing their journey, potentially saving lives.

Additionally, the rise of autonomous vehicles and advanced driver-assistance systems (ADAS) presents new opportunities for reducing the incidence of substance-related accidents. However, the practical implementation of ADAS in developing countries faces several challenges. High costs, outdated vehicle fleets, and limited infrastructure for supporting these technologies make widespread adoption difficult.

Studies indicate that while some emerging economies have begun integrating basic ADAS features, such as automatic emergency braking and lane departure warning, their effectiveness is often hindered by poor road conditions, inconsistent traffic law enforcement, and a lack of standardization in vehicle safety regulations. In contrast, high-income countries benefit from better road infrastructure and regulatory frameworks, enabling more seamless ADAS integration.

Thus, while ADAS holds promise for improving road safety, its adoption in developing nations requires addressing economic and infrastructural limitations before it can have a significant impact on reducing substance-related accidents. Integrating forensic data with technological developments can shape future road safety measures, providing an evidence-based approach to preventing fatal crashes. By expanding the scope of forensic investigations and integrating multidisciplinary expertise, forensic medicine remains a pivotal field in understanding and mitigating road accident fatalities. The combination of external examination, internal autopsy, toxicological analysis, and radiological imaging provides a holistic approach to the investigation of road accident fatalities. This multidisciplinary methodology ensures that all relevant factors are considered, allowing for a thorough assessment of the circumstances surrounding the accident. Forensic investigations not only help to determine the cause of death but also contribute to the development of targeted prevention strategies aimed at reducing road accident mortality. The epidemiological data collected through these investigations are essential for shaping public policy and improving road safety [25].

For example, patterns of injury and substance use identified through autopsy reports can provide crucial insights for designing targeted interventions. While measures such as stricter enforcement of drink-driving laws or public campaigns promoting the responsible use of prescription medications are essential, they alone may not be sufficient. Comprehensive policy changes, improved access to rehabilitation programs, and enhanced post-mortem toxicology screening protocols are also necessary to effectively reduce substance-impaired driving incidents. Additionally, collaborative efforts between forensic experts, law enforcement, and public health authorities can strengthen the impact of these interventions by ensuring evidence-based policymaking and sustained monitoring of trends in impaired driving fatalities Moreover, the findings of forensic investigations can help to identify high-risk populations and geographic areas where additional safety measures are needed, ultimately contributing to a more comprehensive and effective road safety strategy.

## Conclusions

Forensic medical investigations are fundamental in clarifying the circumstances surrounding road accident fatalities. The integration of external examination, autopsy findings, toxicological analysis, and radiological imaging allows forensic experts to reconstruct the dynamics of the accident, assess the role of psychoactive substances, and accurately determine the cause of death. Alcohol remains the most frequently detected substance in fatal road accidents, followed by cannabis and benzodiazepines. Each of these substances presents unique risks to driving performance, and their combined use amplifies the likelihood of severe or fatal crashes. The findings from recent toxicological studies emphasize the persistent problem of impaired driving, despite ongoing prevention campaigns and stricter legal measures. These results highlight the urgent need for improved public awareness programs, expanded routine toxicological screening, and targeted interventions for high-risk populations.

Forensic investigations provide crucial data for identifying trends and risk factors in road traffic accidents, directly informing policy decisions and prevention strategies. Evidence from toxicological analyses has contributed to stricter impaired-driving laws, enhanced drug screening protocols, and targeted public awareness campaigns. To improve road safety, policymakers should prioritize harmonizing regulations, strengthening enforcement, and expanding education initiatives, particularly regarding the impact of prescription medications on driving. A comprehensive, multidisciplinary approach that integrates forensic findings into traffic safety policies is essential for reducing fatalities and enhancing public safety.
